# The Problem of Microbial Dark Matter in Neonatal Sepsis 

**DOI:** 10.3201/eid2611.200004

**Published:** 2020-11

**Authors:** Shamim A. Sinnar, Steven J. Schiff

**Affiliations:** The Pennsylvania State University, University Park, Pennsylvania, USA

**Keywords:** 16S amplicon sequencing, antimicrobial stewardship, bacteria, bloodborne pathogens, cerebral palsy, diagnosis, DNA sequence analysis, Escherichia coli, Group B Streptococcus, Klebsiella, molecular diagnosis, neonatal sepsis, Paenibacillus thiaminolyticus, point of care, polymicrobial infections, RNA sequence analysis, Staphylococcus aureus, total metagenomic sequencing, *Suggested citation for this article*: Sinnar SA, Schiff SJ. The problem of microbial dark matter in neonatal sepsis. Emerg Infect Dis. 2020 Nov [*date cited*]. https://doi.org/10.3201/eid2611.200004

## Abstract

Neonatal sepsis (NS) kills 750,000 infants every year. Effectively treating NS requires timely diagnosis and antimicrobial therapy matched to the causative pathogens, but most blood cultures for suspected NS do not recover a causative pathogen. We refer to these suspected but unidentified pathogens as microbial dark matter. Given these low culture recovery rates, many non–culture-based technologies are being explored to diagnose NS, including PCR, 16S amplicon sequencing, and whole metagenomic sequencing. However, few of these newer technologies are scalable or sustainable globally. To reduce worldwide deaths from NS, one possibility may be performing population-wide pathogen discovery. Because pathogen transmission patterns can vary across space and time, computational models can be built to predict the pathogens responsible for NS by region and season. This approach could help to optimally treat patients, decreasing deaths from NS and increasing antimicrobial stewardship until effective diagnostics that are scalable become available globally.

The term “microbial dark matter” refers to organisms that cannot easily be cultured under available laboratory conditions (*1*). The knowledge that microbial dark matter exists is not new; some of these organisms have been responsible for human infections throughout the history of microbiology. Indeed, Robert Koch himself recognized that the postulates he proposed to demonstrate causality between a microorganism and a disease were not fulfilled in several common diseases, including malaria and leprosy (*2*). One major reason was difficulty in cultivating the responsible organisms. 

Difficult-to-isolate organisms continue to be responsible for serious human infections, such as leptospirosis, syphilis, and many others (*3*,*4*). In fact, organisms that cause even such relatively common and potentially deadly syndromes as neonatal sepsis (NS) often constitute microbial dark matter, not because they cannot in theory be cultured but because in actual cases of NS these organisms are rarely recovered and identified. Organisms that are known to commonly cause NS in some areas of the world include *Escherichia coli*, group B *Streptococcus*, *Klebsiella* spp., and *Staphylococcus aureus* (*5*). However, because in most cases of NS worldwide we do not identify the organisms involved, we often cannot determine optimal treatments for NS or design successful prevention strategies. This problem is compounded by the fact that the organisms known to be frequently associated with NS differ in different parts of the world (*5*,*6*). 

The inability to properly diagnose or treat NS constitutes a substantial global health issue. NS affects »3 million neonates per year worldwide (*7*) and causes »750,000 deaths per year worldwide; rates of death are highest in sub-Saharan Africa (*8*). Children who do survive are at risk for deadly or debilitating sequelae, such as cerebral palsy, seizures, cognitive delays, respiratory disease (*9*), and postinfectious hydrocephalus (*10*,*11*). Thus, timely and effective treatment of NS is imperative to prevent death and reduce sequelae, but when the causative organisms cannot be determined, optimal medical management of NS is problematic. 

## Current State of Diagnosis in NS 

Several studies illustrate the low organism recovery rates from NS cultures. One, a large retrospective study from the United Kingdom, found that the proportion of blood cultures positive for the putative pathogen in individual cases ranged from 0.8% at birth to 15% on day 7 of life (*12*). A recent study from rural Cambodia determined that only 2% of blood cultures from neonates were positive for a pathogen; 10% of those cultures—5 times as many—were positive for likely contaminants (*13*). Similarly, in the recent ANISA (Aetiology of Neonatal Infection in South Asia) study on the causes and incidence of community-acquired serious infections among young children in South Asia, in which blood cultures were obtained in a sterile manner from >4,800 infants with suspected bacterial infection, only 2.1% of cultures were true positives (*14*). 

One reason for the low recovery rates is that, although blood cultures are the gold standard for discovering bacterial pathogens causing NS, their sensitivity can be extremely low (*15*). Blood culturing may fail to identify NS pathogens, in part because very low levels of bacteremia can cause symptoms in neonates (*9*,*16*), and in part because it is difficult to obtain sufficient blood for sensitive culture recovery from small neonates. Furthermore, even if the blood obtained does contain bacteria known to cause NS, these bacteria may not grow well in culture (*17*). In addition, nonbacterial pathogens that do not grow in common culture media can cause NS symptoms; these include viruses such as enteroviruses, rhinoviruses, and coronaviruses (*18*,*19*), fungi such as *Candida* sp. (*19*), and parasites such as *Plasmodium*, the agent responsible for malaria (*20*). Finally, any antimicrobial treatment administered before blood is collected further reduces the chances of recovering pathogens using culturing techniques (*15*). 

This inability to identify pathogens in many cases has serious clinical implications. For most NS cases, clinicians must balance the opposing risks of undertreating a serious bacterial infection or using broad-spectrum antimicrobial drugs that may be unnecessary in many cases. Use of narrow-spectrum antimicrobial drugs without knowing the organisms responsible increases the risk of providing ineffective therapy, which is associated with increased risk for death, infectious complications, and treatment failure (*21*). On the other hand, routinely using broad-spectrum antimicrobial drugs when pathogens cannot be identified can drive antimicrobial resistance (*22*), which runs increasingly high in underresourced areas of the world (*23*). Antimicrobial stewardship is especially important in these communities due to limited access to the newer antimicrobial drugs needed to treat multidrug-resistant bacterial infections (*24*,*25*). 

Moreover, because the epidemiology of NS varies worldwide (*6*), treatment decisions in a particular geographic location cannot be made simply on the basis of the pathogens recovered in other settings. For example, although group B *Streptococcus* is a leading cause of NS in Europe and North America, it does not seem to be a dominant cause of NS in many other regions of the world (*26*). Furthermore, recommendations that culture-negative NS be treated with short courses of narrow spectrum antimicrobials may not be appropriate for the low-resource settings where most NS cases occur (*5*,*22*). 

Another issue complicating diagnosis and treatment is that NS can sometimes have polymicrobial causes (*27*–*29*). Polymicrobial infections tend to be more severe and harder to treat than monomicrobial infections (*27*,*30*). The true rate of polymicrobial NS might be higher than that reported in the studies cited because 1 organism may outcompete the others in culture and because of the detection difficulties detailed. This failure to identify all of the causal organisms may further contribute to inadequate treatment. 

## Limitations of Emerging Technologies for Detecting Pathogens 

Because of the low recovery rate of cultures (*15*), there has been intense interest in developing alternative methods for diagnosing febrile illnesses, including NS (*9*). In theory, some of these newer technologies could be used to more effectively diagnose the causes of NS when a bacterial culture fails to yield results; that is, they could help to address the problem of microbial dark matter in NS diagnosis. All of these methods, however, have critical drawbacks. 

One group of methods relies on targeted PCR to amplify and detect the DNA or RNA of specific organisms from body fluids such as blood or cerebrospinal fluid (CSF) (*31*). However, PCR-based assays can only detect the specific organisms that are targeted by the assay; thus, one cannot discover pathogens that were not expected a priori. This method would be most beneficial when the epidemiology of NS is already well-established for a particular population. A similar principle applies for detecting antimicrobial resistance genes; PCRs can uncover only the resistance genes that they are targeted to detect. Moreover, given that there are now thousands of known antimicrobial resistance genes, it would be very difficult to test for all of these genes individually using PCR, and it remains a challenge for qPCR. Furthermore, novel mutations conferring antimicrobial resistance would still be missed. 

Another approach is 16S amplicon sequencing using PCR to amplify the common 16S ribosomal gene shared by all bacteria. DNA sequencing of this PCR product is then used to identify the specific bacteria (*32*). 16S amplicon techniques can, in theory, uncover bacterial pathogens in an unbiased manner. However, contamination from clinical collection, laboratory reagents, or the laboratory environment can dominate the sequencing results if pathogen sequences in the patient sample are present in very low concentrations (*31*), which is often the case in NS. Bacterial DNA can be present in minute quantities and therefore difficult to detect within the much greater mass of contaminant bacterial DNA acquired during routine sequencing workflows (*32*). 

Methods have been developed to remove some of this contamination, either during the sample preparation process before amplification and sequencing occur (*33*,*34*) or during the computational analysis after sequencing (*35*). In either case, suitable negative and positive controls are crucial for accurate analysis because blood from apparently healthy patients can harbor bacterial sequences (*36*). For NS studies, healthy NS-negative infants from similar environments would be ideal clinical controls, but blood and CSF from healthy infants are rarely sampled. Another control option would be age-matched infants whose blood and CSF samples were taken for reasons unrelated to NS, such as for elective surgery. 

Yet another alternative for pathogen discovery is sequencing the bulk DNA or RNA, or both, contained in the sample. After filtering out the human DNA or RNA, remaining sequences can be analyzed to detect bacteria, viruses, fungi, and parasites (*37*). Some of these sequencing approaches include enrichment steps to increase the detection of viral sequences in an otherwise overwhelming background of host sequences (*38*). Sequencing total DNA and RNA is especially attractive for unbiased discovery of panmicrobial pathogens and can additionally detect polymicrobial infections. In theory, sequencing total DNA and RNA makes it possible to retrieve nucleic acid sequences from all pathogens, regardless of kingdom. Sequencing of total DNA can also detect antimicrobial resistance genes and therefore guide treatment (*39*,*40*). However, sequencing total DNA and RNA has its own challenges, including biases potentially being introduced during sample preparation and by inadequate depth of sequencing, as well as by the current absence of standardized software tools and pipelines for sequence analysis (*41*,*42*). In addition, issues with contamination and lack of suitable negative controls likewise apply to this method. 

Although there are case reports of whole genome sequencing leading to actionable diagnosis in the face of negative blood cultures (*43*,*44*), these methods are not yet ready to be implemented in routine diagnostics. The more complex sequencing techniques, though effective in certain cases, remain research laboratory endeavors at the moment. Even if some of these sequencing-based assays could be incorporated into a diagnostic workflow, the high cost, technical optimization required, and bioinformatic and statistical expertise needed to analyze complex sequencing data currently make this technology impractical, even in high-resource settings. Therefore, unbiased pathogen discovery technology is at present neither scalable nor sustainable on a global level and remains difficult to implement effectively in the resource-poor countries where incidence and death from NS are highest. 

## Moving Forward in Neonatal Sepsis Diagnosis 

Until lower-cost solutions can be disseminated, pathogen discovery requiring costly and sophisticated technology might need to be performed at research laboratories or specialized institutions remote from clinical centers, which means that discovery cannot be done effectively in real time. Therefore, reducing neonatal death worldwide from infections caused by microbial dark matter might require a paradigm shift. 

Population-wide discovery of NS pathogens may be a way forward. Hundreds of blood or CSF samples with proper controls can be collected from regional treatment sites and analyzed by sophisticated sequencing methods at separate pathogen discovery centers. Population-wide results defining the probabilistic distributions of likely pathogens by location could then be used to inform the most effective treatment strategies, including antimicrobial choices, at the point of care. In other words, clinicians would better know which organisms would be the most likely to cause NS in their patient population and, therefore, which antimicrobials from their often limited choices would most effectively treat the NS. Such knowledge would increase clinicians’ ability to optimize treatment and reduce illness and death from NS. We call this approach predictive personalized public health. 

If pathogen transmission patterns were stable across time, discovering the organisms causing NS would only need to be done once. However, the pathogens responsible for NS may differ by season, akin to the seasonally varying distribution of pathogens responsible for diseases such as cholera, malaria, and melioidosis (*45*–*47*). Therefore, being able to predict the pathogens most likely responsible for NS, or any other syndromic disease, based on the season as well as the patient’s geographic location would be ideal. Predictive computational models of the diseases in question could be developed to identify the most likely pathogens for a specific region at a specific time of year. Combined with data on a pathogen’s resistance to specific antimicrobial drugs, this approach could optimize the use of broad- versus narrow-spectrum antimicrobial drugs to improve both treatment outcomes and antimicrobial stewardship. 

The feasibility of initiating such an organism discovery and modeling approach has been recently shown in Uganda. Over the past few decades, in thousands of cases of infant postinfectious hydrocephalus resulting from neonatal sepsis, CSF samples have failed to yield any positive cultures (*10*; S.J. Schiff, unpub. data). However, more recently, advanced genomic techniques were used to identify a difficult-to-culture novel bacterial strain, *Paenibacillus thiaminolyticus* Mbale (*48*), and demonstrate frequent co-infection with human herpesvirus 5 (cytomegalovirus) in many of these infants with postinfectious hydrocephalus (*49*). Spatial GPS data demonstrated that, compared to control cases, the bacterial infections were localized to the swampy regions north and south of the banks of Lake Kyoga (p<0.03), whereas the cytomegalovirus infections were distributed widely (*49*). The statistically significant spatial discrimination of infection locations by GPS demonstrated in these cases, combined with an established association of rainfall with infant postinfectious hydrocephalus in Uganda (*50*), highlights the substantial potential for predictive models to optimize point-of-care treatment. 

In conclusion, for syndromic illnesses such as NS that are not linked to a specific pathogen, effective diagnostics to identify microbial dark matter and guide treatment are urgently needed. However, until point-of-care molecular diagnostics can be sustainably implemented in regions of the world with the greatest disease burden, alternative predictive treatment models such as the one we described may help to reduce illness and death. 
